# Remote Sensing Imagery Data Analysis Using Marine Predators Algorithm with Deep Learning for Food Crop Classification

**DOI:** 10.3390/biomimetics8070535

**Published:** 2023-11-10

**Authors:** Ahmed S. Almasoud, Hanan Abdullah Mengash, Muhammad Kashif Saeed, Faiz Abdullah Alotaibi, Kamal M. Othman, Ahmed Mahmud

**Affiliations:** 1 Department of Information Systems, College of Computer and Information Sciences, Prince Sultan University, Riyadh 11586, Saudi Arabia; almasoud@psu.edu.sa; 2 Department of Information Systems, College of Computer and Information Sciences, Princess Nourah bint Abdulrahman University, Riyadh 11671, Saudi Arabia; 3 Department of Computer Science, Applied College, Muhayil, King Khalid University, Abha 61421, Saudi Arabia; 4 Department of Information Science, College of Humanities and Social Sciences, King Saud University, Riyadh 11437, Saudi Arabia; 5 Department of Electrical Engineering, College of Engineering and Islamic Architecture, Umm Al-Qura University, Makkah 21955, Saudi Arabia; kmothman@uqu.edu.sa; 6 Research Center, Future University in Egypt, New Cairo 11835, Egypt

**Keywords:** remote sensing images, deep learning, crop classification, machine learning, computer vision

## Abstract

Recently, the usage of remote sensing (RS) data attained from unmanned aerial vehicles (UAV) or satellite imagery has become increasingly popular for crop classification processes, namely soil classification, crop mapping, or yield prediction. Food crop classification using RS images (RSI) is a significant application of RS technology in agriculture. It involves the use of satellite or aerial imagery to identify and classify different types of food crops grown in a specific area. This information can be valuable for crop monitoring, yield estimation, and land management. Meeting the criteria for analyzing these data requires increasingly sophisticated methods and artificial intelligence (AI) technologies provide the necessary support. Due to the heterogeneity and fragmentation of crop planting, typical classification approaches have a lower classification performance. However, the DL technique can detect and categorize crop types effectively and has a stronger feature extraction capability. In this aspect, this study designed a new remote sensing imagery data analysis using the marine predators algorithm with deep learning for food crop classification (RSMPA-DLFCC) technique. The RSMPA-DLFCC technique mainly investigates the RS data and determines the variety of food crops. In the RSMPA-DLFCC technique, the SimAM-EfficientNet model is utilized for the feature extraction process. The MPA is applied for the optimal hyperparameter selection process in order to optimize the accuracy of SimAM-EfficientNet architecture. MPA, inspired by the foraging behaviors of marine predators, perceptively explores hyperparameter configurations to optimize the hyperparameters, thereby improving the classification accuracy and generalization capabilities. For crop type detection and classification, an extreme learning machine (ELM) model can be used. The simulation analysis of the RSMPA-DLFCC technique is performed on two benchmark datasets. The extensive analysis of the results portrayed the higher performance of the RSMPA-DLFCC approach over existing DL techniques.

## 1. Introduction

Recent developments in remote sensing (RS) data and technologies deliver the ability of highly accessible, cheap and real time advantages [1]. In recent years, a massive quantity of global coverage RS images have been openly available [2]. In particular, Landsat 8 satellite offers high-resolution multispectral datasets including wealthy data on agricultural vegetation development which is easily accessible. It allows us to examine the vegetation growth and forecast the changes over time from past to present [3]. RS is an effective data collection technology, and it is broadly employed in agriculture, for example, to monitor crop conditions, crop distribution, and to predict upcoming food production under various situations [4]. Though current agricultural RSs generally use sensors from satellite environments like Landsat and MODIS, they combine and integrate the data acquired from the aerial or ground-based sensors [5,6]. Even if satellite-borne sensors cover a larger range from a local to a national scale, precision agriculture needs remotely sensed data with high efficiency, knowledge, and high resolution to sufficiently study crop conditions, hence giving support to national food provision security. 

Aerial or airborne RS that uses classical aerial photography taken from aircraft, light aircraft or unmanned aerial vehicles (UAVs) as its platform, and gets a higher ground resolution of a few centimeters than the satellite image resolution of a few to hundreds of meters provides two important advantages: Primarily, significant biochemical and biophysical variables can be calculated finely at most of the levels of an individual plant, and its images are without mixed pixel effects. Next, important phases of crop development can be finely noticed with the use of active and current crop height created by classical aerial triangulation technology [7]. Additionally, the highly accurate cropland mask, crop-specific categorization and circulation gained from airborne sensors provide extra training and validation data for satellite observation and additionally increase the respective outcome. Successful integration of various sensor sources, wavebands, and time-stamped RS images gives extensive feature data about crops [8]. Thus, it is a reasonable and significant study to discover the crop classification based on RS images. 

Classical RS-based image classification procedures of ML were slowly used in the classification and detection of RS images. These models can be classified as supervised and unsupervised classes. The first holds minimum distance, maximum likelihood, and support vector machine (SVM). In this phase, the SVM is extensively applied in RS image classification, even though few problems exist. DL, referring to a deep neural network, is a type of ML technique, and because of its data expression and dominant feature extraction capability, it has been widely adopted. Over the years, the identification rate of DL on most classical identification processes has enhanced considerably [9]. Numerous studies have exhibited that DL can extract features from RS imagery and enhance the classifier performance. 

This article develops a remote sensing imagery data analysis using the marine predators algorithm with deep learning for food crop classification (RSMPA-DLFCC) method. The RSMPA-DLFCC technique mainly investigates the RS data and determines the variety of food crops. In the RSMPA-DLFCC technique, the SimAM-EfficientNet model is utilized for the feature extraction process. The MPA is applied for the optimal parameter selection to optimize the accuracy of SimAM-EfficientNet architecture. MPA, inspired by the foraging behaviors of marine predators, perceptively explores hyperparameter configurations to optimize the hyperparameters, thereby improving the classification accuracy and generalization capabilities. For crop type detection and classification, an extreme learning machine (ELM) model can be used. The simulation analysis of the RSMPA-DLFCC method takes place on the UAV image dataset. 

The rest of the paper is organized as follows. Section 2 provides the related works and Section 3 offers the proposed model. Then, Section 4 gives the result analysis and Section 5 concludes the paper.

## 2. Literature Review

Kwak and Park [10] examined self training with domain adversarial networks (STDAN) to classify crop types. The main function of STDAN is to integrate adversarial training for improving spectral discrepancy issues with self training in order to create novel trained data in the targeted field, utilizing present ground truth details. In [11], a unique structure based on deep CNN (DCNN) and the dual attention module (DAM) makes utilization of the Sentinel 2 time series dataset which was projected for crop identification. Fresh DAM was applied to the removal of enlightened deep features using the advantages of spatial and spectral features of Sentinel 2 datasets. Reedha et al. [12] targeted the design of attention-related DL networks in a significant technique to state the earlier mentioned complications regarding weeds and crop detection with drone systems. The objective is to inspect visual transformers (ViT) and implement them in the identification of plants in UAV images. In [13], the results of accurate recognition were tested to associate the phenology of vegetation products by time series of Landsat8, digital elevation model (DEM), and Sentinel 1. Next, based on the agricultural phenology of crops, radar Sentinel1 and optical Landsat8 time-series data with DEM were used to enhance the performance classification. 

Sun et al. [14] proposed a technique for attaining deduction of fine-scale crops by combining RS information from different satellite images by construction of chronological scale crop features inside the parcels employing Sentinel 2A, Gaofen-6, and Landsat 8. The authors adopted a feature-equivalent technique to fill in the missing values in the time series feature-building methods to prevent problems with unidentified crops. Li et al. [15] introduced a scale sequence object-based CNN (SS-OCNN) that identifies images at the object phase by taking segmented object crop parcels as the primary unit of analysis, therefore providing the limits between crop parcels that were defined precisely. Next, the segmented object was identified utilizing the CNN approach combined with an automated generating scale structure of input patch sizes.

Zhai et al. [16] examined the contribution of the data to rice planting area mapping. Specifically, the introduction of the red-edge band was to build a red-edge agricultural index derived from Sentinel 2 data. C band quad pol Radar sat 2 data was also utilized. The authors employed the random forest technique and finally collaborated with radar and optical data to plot rice-planted regions. In [17], the authors designed an enhanced crop planting structure to plot the structure for rainy and cloudy regions using collective optical data and SAR data. First, the author removed geo parcels from optical images with high dimensional resolution. Next, the authors made an RNN-based classification appropriate for remote detecting images on a geo parcel scale. 

## 3. The Proposed Model

**Figure 1:**
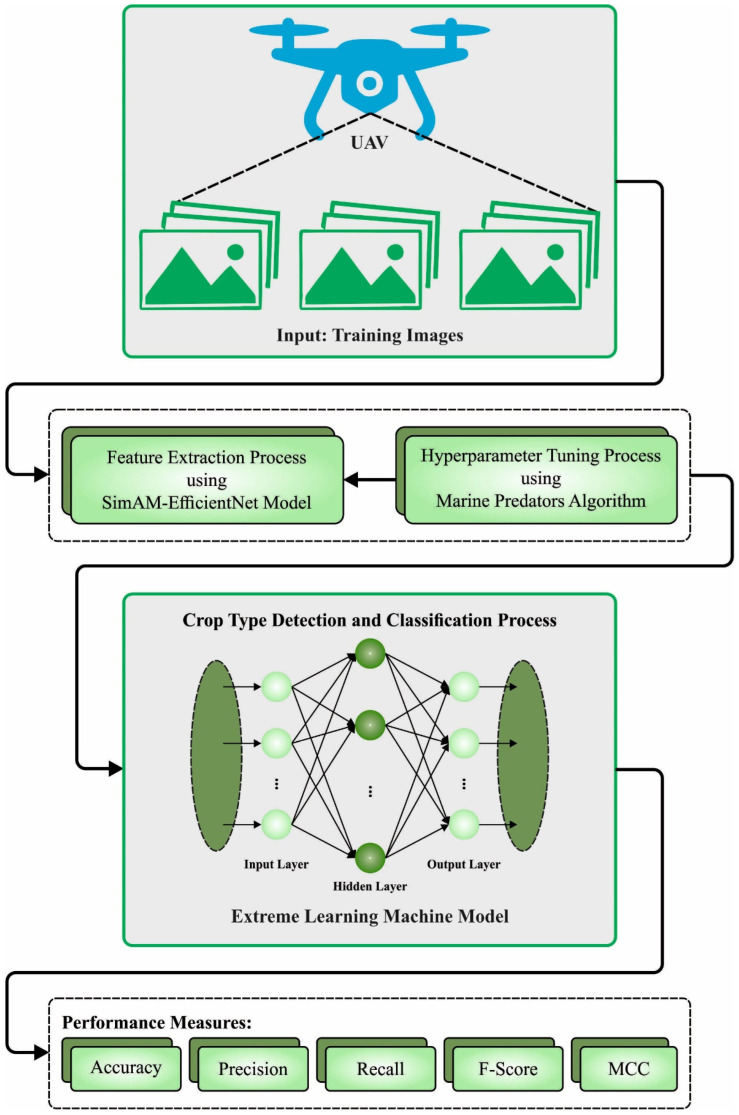
Overall process of RSMPA-DLFCC algorithm.

This manuscript offered the development of automated food crop classification using the RSMPA-DLFCC technique. The RSMPA-DLFCC technique mainly investigates the RS data and determines different types of food crops. In the RSMPA-DLFCC technique, three major phases of operations are involved, namely the SimAM-EfficientNet feature extractor, MPA-based hyperparameter tuning, and ELM classification. Figure 1 represents the entire process of the RSMPA-DLFCC approach.

### 3.1. Feature Extraction Using SimAM-EfficientNet Model

The RSMPA-DLFCC technique applies the SimAM-EfficientNet model to derive feature vectors. A novel CNN called EfficientNet was launched by Google researchers [18]. The study uses a multi-dimensional hybrid method scaling model making them consider the speed and accuracy of the model even though the existing network has advanced considerably in speed and accuracy. Through compound scaling factors, ResNet raises the network depth to optimize the performance. By improving accuracy and ensuring speed, EfficientNet balances the network depth, width, and resolution. EfficientNet-B0 is the initial EfficientNet model. The most basic model B0 is: concerning resolution, layers, and channels, B1-B7 overall of 7 models adapted from B0.

**Figure 2:**
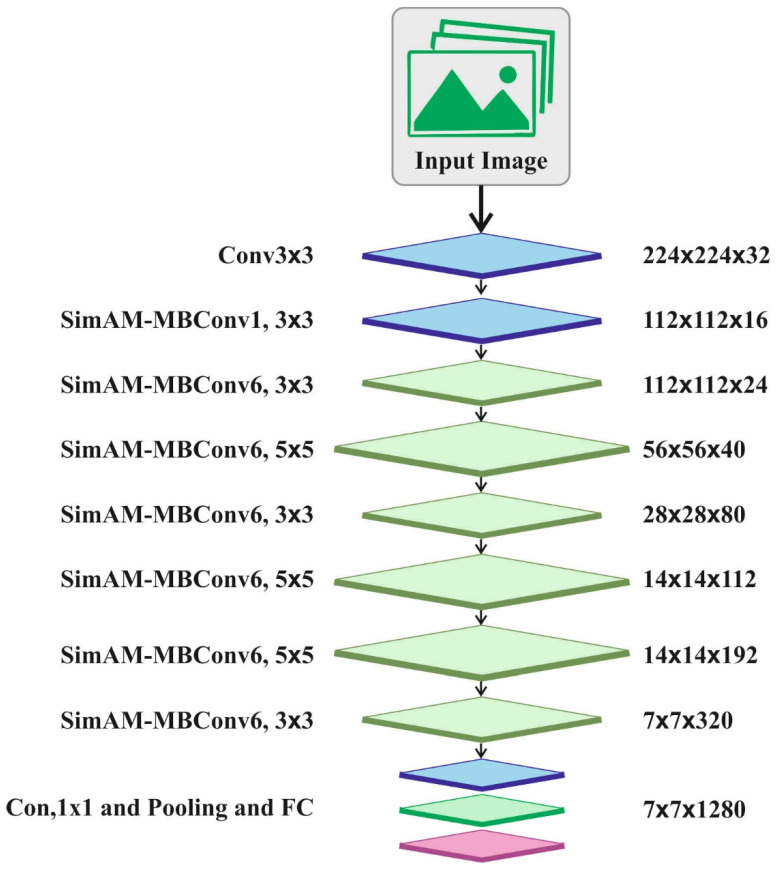
Architecture of SimAM-EfficientNet.

Many existing attention modules generate 1D or 2D weights. Next, the weights created are extended for channel and spatial attention. Generally, the present attention module faces the two subsequent challenges. The former is the attention module could extract features through channel and space that results in the flexibility of attention weight. Moreover, CNN is influenced by a series of factors and has a complex structure. SimAM considers these spaces and channels in contrast to them. Without adding parameters, it presents 3D attention weights to the original network. Based on neuroscience theory, an energy function can be defined and, in turn, derive a solution that converges faster. This operation is executed in ten lines of code. An additional benefit of SimAM is that it prevents excessive adjustment to the network architecture. Hence, SimAM is lightweight, more flexible, and modular. In numerous instances, SimAM is better than the conventional CBAM and SE attention models. Figure 2 illustrates the architecture of SimAM-EfficientNet.

The SimAM model defines an energy function and looks for important neurons. It adds regular terms and uses binary labels. At last, the minimal energy is evaluated by the following expression:

(1) $$e_{t}^{*}=\left(4\left(\lambda +\sigma^{2}\right)\right)/(\left(t-u{)}^{2}+2\sigma^{2}+2\lambda \right)\;$$

(2) $$u_{t}=\frac{1}{M-1}\overset{M-1}{\underset{i=1}{\sum }}x_{i},\sigma_{t}^{2}=\frac{1}{M-1}\overset{M-1}{\underset{i=1}{\sum }}{\left(x_{i}-u_{t}\right)}^{2}\;$$

where $\mu_{t}$ and $\sigma_{t}^{2}$ are the mean and variance of each neuron. t is the target neuron. λ indicates the regularization coefficient. Using M=H×W, the neuron count on that channel is attained. Finally, the dissimilarity between neurons and peripheral neurons is associated with the energy used. The implication of all the neurons is evaluated by $1/e^{*}$. The scaling operator is used to refine the feature and it can be formulated as follows:

(3) X=X·sigmoid (1/E) 

The sigmoid function is used to limit the size of the E value. In Equation (3), E group each e across the channel and spatial sizes. 

EfficientNet-B0 has a total of nine phases. The initial phase is 3×3 convolutional layers. The second to the eighth phases are MBConv, which is the building block of these network models. The last phase is made up of a pooling layer, a 1×1 convolutional layer, and the FC layer. MBConv has five different parts. The initial part is a 1×1 convolutional layer. The next part is a depth-wise convolution layer. The third part is the SE attention mechanism. The fourth part is a 1×1 convolutional layer for reduction dimension. Lastly, the dropout layer lessens the over-fitting problem. After the first convolutional layer, the SimAM module was added to increase channel and spatial weights. The original EfficientNet comprises the SE attention mechanism. 

The SimAM-EfficientNet is made up of seven SimAM-MBConv models, one FC layer, two convolution layers, and one pooling layer. At first, the images with 224×224×3 dimensions are ascended by the 3×3 convolution layers. The dimensions of the images obtained with features are 112×112×32. Next, the image features are extracted by the SimAM-Conv. The connection will be deactivated when both SimAM-Convs are the same, and the input will connect. The FC layer is utilized for classification and the original channel is restored after a 1×1 point-wise convolutional layer.

### 3.2. Hyperparameter Tuning Using MPA

For the optimal hyperparameter selection process, the MPA is applied. The MPA is derived from the foraging tactics of the ocean predator [19]. MPA is a population-based metaheuristic approach. The optimization technique begins with the arbitrary solution.

(4) $$X_{0}=X_{\min\;}+rand\left(X_{\max\;}-X_{\min\;}\right)$$

where $X_{\min\;}$ and $X_{\max}$ denotes the lower and upper boundaries, and rand is a randomly generated integer in the range [0,1]. In the MPA, Prey and Elite are two different matrices with similar dimensions. The optimum solution is selected as the fittest predator while creating the Elite matrix.

The finding of and search for prey is checked through these matrices. ${\vec{X}}^{I}$ indicates the dominant predator vector, n is the searching agent, and d, the dimension. Both prey and predator are the search agents.

(5) $$Elite={\left[\begin{array}{cccc} X_{1,1}^{I} & X_{1,2}^{I} & \ldots & X_{1,d}^{I}\\ X_{2,1}^{I} & X_{2,2}^{I} & \ldots & X_{2,d}^{I}\\ \vdots & \vdots & \vdots & \vdots \\ \vdots & \vdots & \vdots & \vdots \\ X_{n,1}^{I} & X_{n,2}^{I} & \ldots & X_{n,d}^{I} \end{array}\right]}_{nxd}\;\;\;\;$$

where $the\;j^{th}$ dimension of $i^{th}$ prey is represented as $X_{i,j}$. The optimization method is connected to both matrices. Predator uses these matrices for updating the position.

(6) $$Prey={\left[\begin{array}{cccc} X_{1,1} & X_{1,2} & \ldots & X_{1,d}\\ X_{2,1} & X_{2,2} & \ldots & X_{2,d}\\ X_{3,1} & X_{3,1} & \ldots & X_{3,d}\\ \vdots & \vdots & \vdots & \vdots \\ \vdots & \vdots & \vdots & \vdots \\ X_{n,1} & X_{n,2} & \ldots & X_{n,d} \end{array}\right]}_{nxd}$$

In the MPA, there are three stages discussed in detail.

Phase 1 occurs if $<\left(\left({\mathrm{Max}}_{-}Iter\right)/3\right)$. Iter and ${\mathrm{Max}}_{-}Iter$ denote the existing and maximal iteration counter. P shows the constant number with the value of 0.5. The appropriate tactic is one where the predator should stop. In Equation (7) of stage 1, vector $R_{B}$ portrays the Brownian motion and uniformly distributed random number in [0,1].

(7) $${\vec{stepsize}}_{i}=\vec{R}⊗\left({\vec{Elite}}_{i}-{\vec{R}}_{B}⊗{\vec{Prey}}_{i}\right)i=1,\;\ldots n\;\;\;{\vec{Prey}}_{i}={\vec{Prey}}_{i}+P\cdot \vec{R}⊗{\vec{stepsize}}_{i}$$

Phase 2 realized if $\left(\left({\mathrm{Max}}_{-}Iter\right)/3\right)<Iter<(\left(2{\mathrm{Max}}_{-}Iter\right)/3$. Once the prey movement is Lévy, then the predator movement should be Brownian. The prey is responsible for exploitation, and the predator is responsible for exploration. The multiplication of ${\vec{R}}_{L}$ and Prey represent the prey movement, and the prey movement can be exemplified by adding the stepsize to the prey position. The ${\vec{R}}_{L}$ vector is a random number representing Lévy motion. CF denotes an adaptive parameter. stepsize for the predator movement can be controlled by the CF.

(8) $${\vec{stepsize}}_{i}={\vec{R}}_{L}⊗\left({\vec{Elite}}_{i}-{\vec{R}}_{L}⊗{\vec{Prey}}_{i}\right)i=1,\;\ldots n/2\;{\vec{Prey}}_{i}={\vec{Prey}}_{i}+P\cdot \vec{R}⊗{\vec{stepsize}}_{i}$$

(9) $${\vec{stepsize}}_{i}={\vec{R}}_{B}⊗\left({\vec{R}}_{B}⊗{\vec{Elite}}_{i}-{\vec{Prey}}_{i}\right)i=\frac{n}{2},\;\ldots n\;{\vec{Prey}}_{i}={\vec{Elite}}_{i}+P\cdot CF⊗{\vec{stepsize}}_{i}\;CP={\left(1-\frac{Iter}{Max_{-}Iier}\right)}^{\left(2\frac{lter}{Max_{-}Iter}\right)}\;$$

Phase 3 occurs If $>\left(\left(2{\mathrm{Max}}_{-}Iter\right)/3\right)$. As the optimum strategy, the predator movement is Lévy.

(10) $${\vec{stepsize}}_{i}={\vec{R}}_{L}⊗\left({\vec{R}}_{L}⊗{\vec{Elite}}_{i}-{\vec{Prey}}_{i}\right)i=1,\;\ldots n\;{\vec{Prey}}_{i}={\vec{Elite}}_{i}+P\cdot CF⊗{\vec{stepsize}}_{i}$$

The factors including fish aggregating devices (FADs) or eddy formation may affect the predator strategy are called the FADs effect. r is a randomly generated value within [0,1]. $\vec{\;U}$ shows the binary vector with an array of 0 and 1. r1 and r2 depict the random indexes of prey matrices. ${\vec{X}}_{\min}$ and ${\vec{X}}_{max}$ denote the lower and upper boundaries of the dimension.

(11) $${\vec{Prey}}_{i}=\{\begin{array}{l} {\vec{Prey}}_{i}+CP\left[{\vec{X}}_{min}+\vec{R}⊗\left({\vec{x}}_{max}-{\vec{X}}_{min}\right)\right]⊗\vec{U},\;r\leq FADs\\ {\vec{Prey}}_{i}+\left[FADs\left(1-r\right)+r\right]\left({\vec{Prey}}_{i}-{\vec{Prey}}_{i}\right),\;r>FADs \end{array}\;$$

The fitness selection is a major factor in the MPA technique. An encoded solution is used for evaluating the outcome of the solution candidate. The accuracy values are the foremost conditions used to design an FF.

(12) Fitness= max (P)

(13) $$P=\frac{TP}{TP+FP}$$

where TP and FP represent the true and false positive values.

### 3.3. Classification Using ELM Model

The ELM algorithm is applied for the automated detection and classification of food crops. The ELM model is used to generate the weight between the hidden and the input layers at random, and during the training process, it does not need to be adjusted and only needs to set the number of HL neurons in order to attain an optimum result [20]. Assume N arbitrary sample (X, t), where $X_{j}=[x_{j1},\;x_{j2}\ldots x_{jn}{]}^{T}\in R^{n},t_{i}=[t_{i1},\;t_{i2}\ldots t_{im}{]}^{T}\in$ R is formulated by

(14) $$\overset{L}{\underset{i=1}{\sum }}\beta_{i}g\left(W_{i}\cdot X_{j}+b_{i}\right)=t_{j},j=1,\ldots ,N$$

The weight of $i^{th}$ neurons in the input layer and HL is $W_{i}={\left[w_{i1},\;w_{i2}\ldots w_{in}\right]}^{T}$, chosen at random. The resultant weight is $\beta_{i}$, and the learning objective is to obtain the fittest $\beta_{i}.$ The $j^{th}$ input vector is $X_{j}$. The inner product of $W_{i}$ and $X_{j}$ is $W_{i}\cdot X_{j}.$ The bias of $i^{th}$ HL neuron is $b_{i}$. The set non-linear activation function is g(x). The output vector of the $i^{th}$ neurons is $g\left(W_{i}\cdot X_{j}+b_{i}\right)$. The target vector attained from the $j^{th}$ input vector is $t_{j}$. It can be represented in the matrix form:

Hβ=T

$$H\left(W_{1},\;\ldots ,\;W_{L},\;b_{1},\;\ldots ,\;b_{L},\;\ldots ,\;X_{1},\;\ldots ,\;X_{L}\right)$$

$$=\left[\begin{array}{ccc} g\left(W_{1}\cdot X_{1}+b_{1}\right) & \ldots & g\left(W_{L}\cdot X_{N}+b_{1}\right)\\ \vdots & \ddots & \vdots \\ g\left(W_{1}\cdot X_{N}+b_{1}\right) & \ldots & g\left(W_{L}\cdot X_{N}+b_{1}\right) \end{array}\right]$$

(15) $$\beta =\left[\begin{array}{c} \beta_{1}^{T}\\ \vdots \\ \beta_{L}^{T} \end{array}\right]\;and\;T=\left[\begin{array}{c} T_{1}^{T}\\ \vdots \\ T_{L}^{T} \end{array}\right]\;$$

The output of the HL node is H, the output weight is β, and the desired output is T. The following equation is used to get ${\hat{W}}_{i},{\hat{\beta }}_{i},{\hat{\;b}}_{i}$ as follows:

(16) $$\left\|H\left({\hat{W}}_{i},{\hat{b}}_{i}\right){\hat{\beta }}_{i}-T\right\|=\underset{W,b,\beta }{\min}\|H\left(w_{i},\;b_{i}\right)\beta_{i}-T\|,\;i=1,\ldots ,L$$

As shown in Equation (17), this corresponds to minimalizing the loss function,

(17) $$E=\overset{N}{\underset{j=1}{\sum }}{\left(\overset{L}{\underset{i=1}{\sum }}\beta_{i}g\left(W_{i}\cdot X_{j}+b_{i}\right)-t_{j}\right)}^{2}$$

Since the HL offset and the input weight $W_{i}$ are determined randomly, then the output matrix of HL is also defined. As shown in Equation (18), the training purpose is transmuted into resolving a linear formula Hβ=T:

(18) $$\hat{\beta }=H^{+}T$$

where the optimum output weight is $\hat{\beta }$. The Moore–Penrose generalized the inverse of H matrix is $H^{+}$, and it is shown that the norm of the obtained solution is unique and minimal. Thus, ELM has better robustness and generalization. 

## 4. Results Analysis 

The proposed model is simulated using the Python 3.8.5 tool. The proposed model is experimented on PC i5-8600k, GeForce 1050Ti 4 GB, 16 GB RAM, 250 GB SSD, and 1 TB HDD. The food crop classification performance of the RSMPA-DLFCC system is validated on the UAV image dataset [21], comprising 6450 samples with six classes. For experimental validation, we have used 80:20 and 70:30 of training (TR)/testing (TS) set.

**Figure 3:**
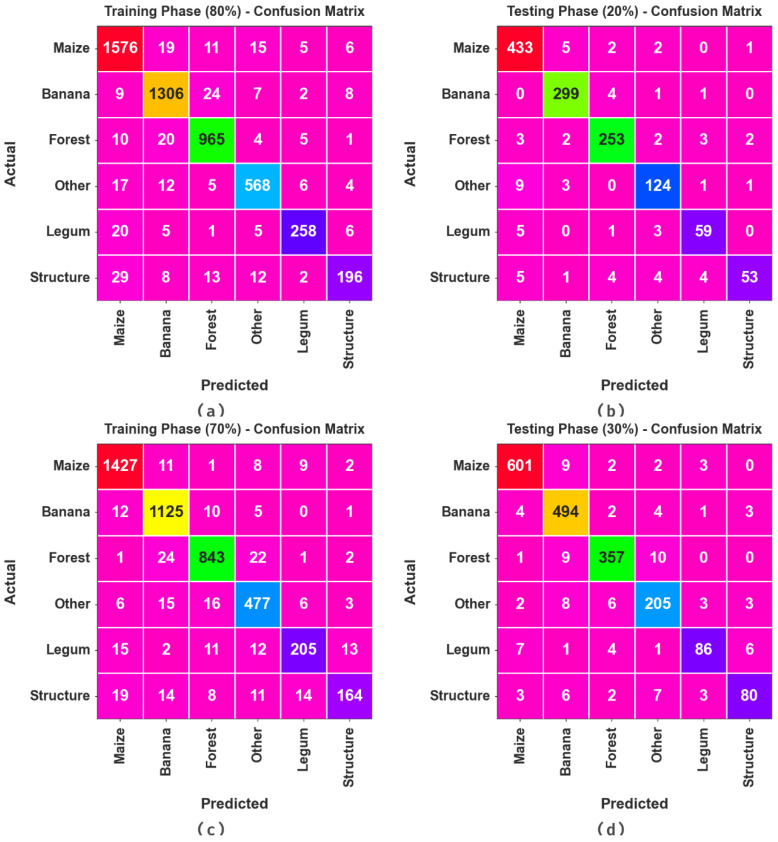
Confusion matrices of (**a**,**b**) 80:20 of TR phase/TS phase and (**c**,**d**) 70:30 of TR phase/TS phase.

Figure 3 demonstrates the confusion matrices produced by the RSMPA-DLFCC technique under 80:20 and 70:30 of the TR phase/TS phase. The experimental values specified the efficient recognition of all six classes.

**Figure 4:**
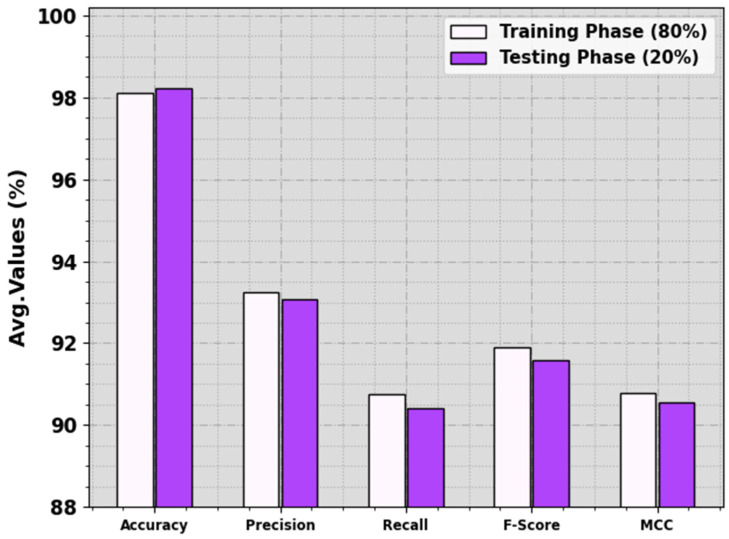
Average of RSMPA-DLFCC algorithm at 80:20 of TR phase/TS phase.

In Table 1 and Figure 4, the food crop classification analysis of the RSMPA-DLFCC methodology is calculated at 80:20 of the TR phase/TS phase. The observational data specified that the RSMPA-DLFCC system properly categorizes seven types of crops. With 80% of the TR phase, the RSMPA-DLFCC technique offers an average $accu_{y}$ of 98.12%, $prec_{n}$ of 93.23%, $reca_{l}$ of 90.76%, $F_{score}$ of 91.89%, and MCC of 90.77%. Additionally, with 20% of TS phase, the RSMPA-DLFCC method offers an average $accu_{y}$ of 98.22%, $prec_{n}$ of 93.06%, $reca_{l}$ of 90.42%, $F_{score}$ of 91.57%, and MCC of 90.56%, respectively.

**Figure 5:**
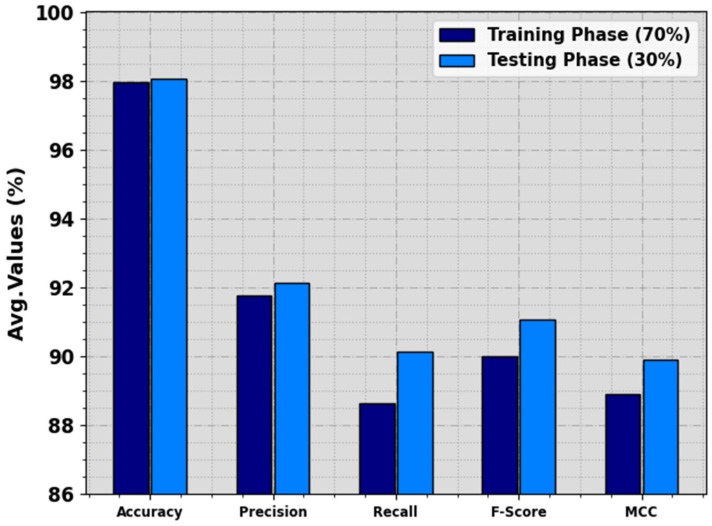
Average of RSMPA-DLFCC algorithm at 70:30 of TR phase/TS phase.

In Table 2 and Figure 5, the food crop classification analysis of the RSMPA-DLFCC technique is calculated at 70:30 of TR Phase/TS Phase. The experimental values indicate that the RSMPA-DLFCC technique appropriately categorizes seven types of crops. With 70% of the TR phase, the RSMPA-DLFCC algorithm offers an average $accu_{y}$ of 97.98%, $prec_{n}$ of 91.79%, $reca_{l}$ of 88.64%, $F_{score}$ of 90.02%, and MCC of 88.90%, respectively. In addition, with 30% of TS phase, the RSMPA-DLFCC system offers average $accu_{y}$ of 98.07%, $prec_{n}$ of 92.13%, $reca_{l}$ of 90.13%, $F_{score}$ of 91.06%, and MCC of 89.92%, correspondingly.

**Figure 6:**
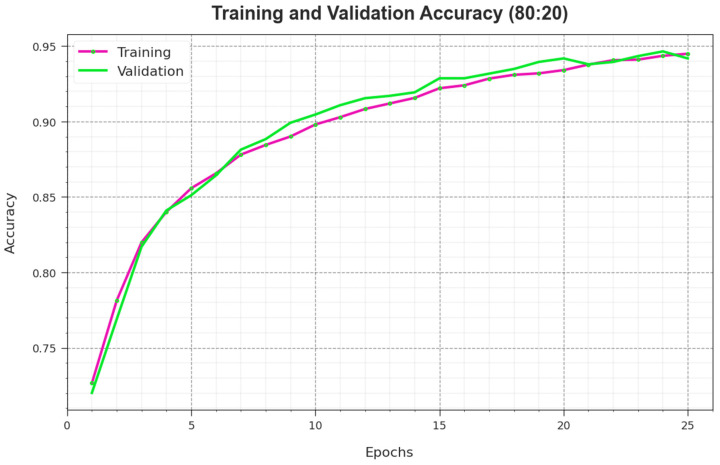
*Accu_y_* curve of RSMPA-DLFCC algorithm at 80:20 of TR phase/TS phase.

To calculate the performance of the RSMPA-DLFCC methodology on 80:20 of TR Phase/TS Phase, TR and TS $accu_{y}$ curves are defined, as shown in Figure 6. The TR and TS $accu_{y}$ curves demonstrate the performance of the RSMPA-DLFCC technique over numerous epochs. The figure offers the details about the learning task and generalization capabilities of the RSMPA-DLFCC system. With a rise in epoch count, it is observed that the TR and TS $accu_{y}$ curves attained are enhanced. It is noted that the RSMPA-DLFCC approach enriches testing accuracy that has the ability to identify the patterns in the TR and TS data.

**Figure 7:**
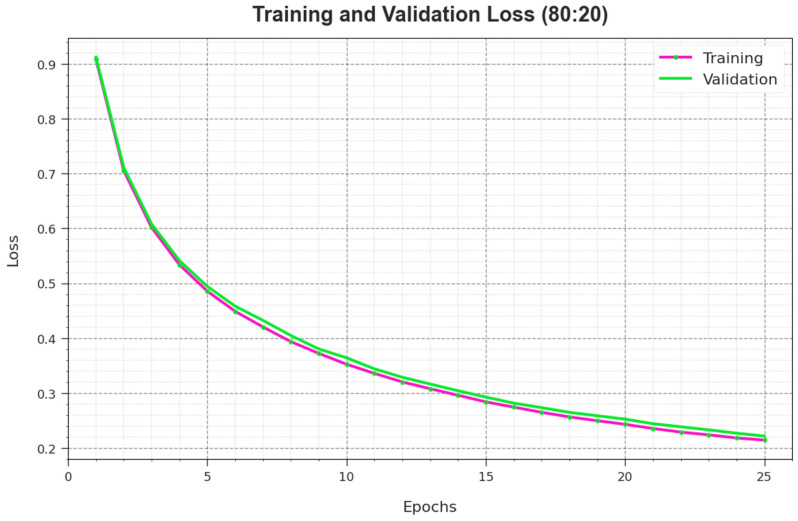
Loss curve of RSMPA-DLFCC algorithm at 80:20 of TR phase/TS phase.

Figure 7 illustrates an overall TR and TS loss value of the RSMPA-DLFCC methodology on 80:20 of TR Phase/TS Phase over epochs. The TR loss shows the model loss acquired reduces over epochs. Mainly, the loss values are decreased as the model adapts the weight to diminish the predicted error on the TR and TS data. The loss analysis illustrates the level where the model is fitting the training data. It is evidenced that the TR and TS loss is progressively minimized and described that the RSMPA-DLFCC technique effectively learns the patterns revealed in the TR and TS data. It is also observed that the RSMPA-DLFCC methodology modifies the parameters for reducing the difference between the predicted and actual training labels.

**Figure 8:**
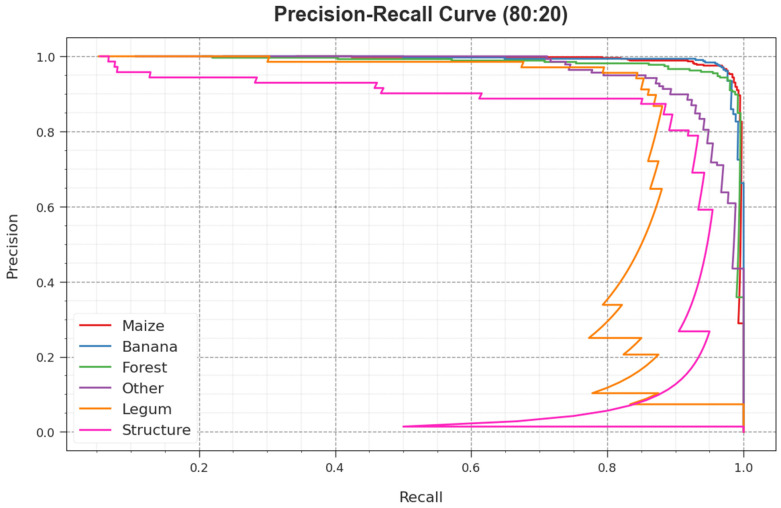
PR curve of RSMPA-DLFCC algorithm at 80:20 of TR/TS phase.

The PR curve of the RSMPA-DLFCC approach on 80:20 of TR phase/TS phase, illustrated by plotting precision against recall as described in Figure 8, confirms that the RSMPA-DLFCC technique achieves improved PR values under all classes. The figure represents that the model learns to identify different class labels. The RSMPA-DLFCC achieves improved effectiveness in the recognition of positive samples with reduced false positives. 

**Figure 9:**
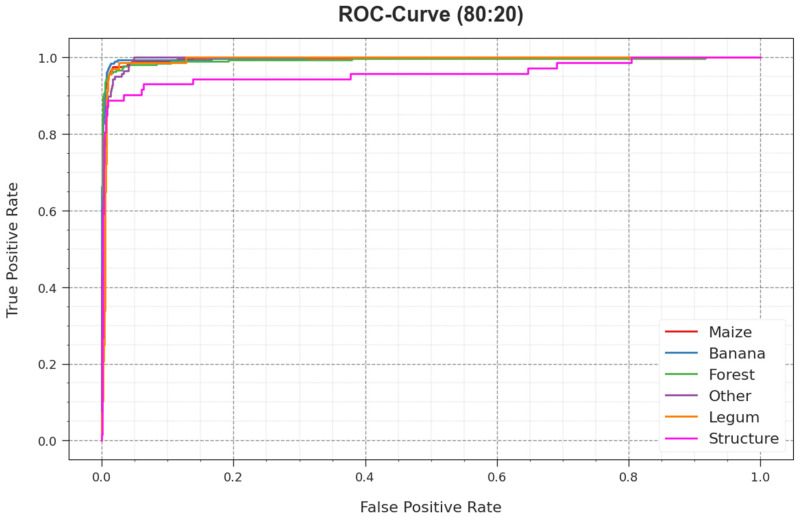
ROC curve of RSMPA-DLFCC algorithm at 80:20 of TR/TS phase.

The ROC analysis, provided by the RSMPA-DLFCC system on 80:20 of TR phase/TS phase demonstrated in Figure 9, has the ability the differentiate between class labels. The figure shows valuable insights into the trade-off between the TPR and FPR rates over dissimilar classification thresholds and differing numbers of epochs. It introduces the accurately predicted performance of the RSMPA-DLFCC methodology on the classification of various classes.

**Figure 10:**
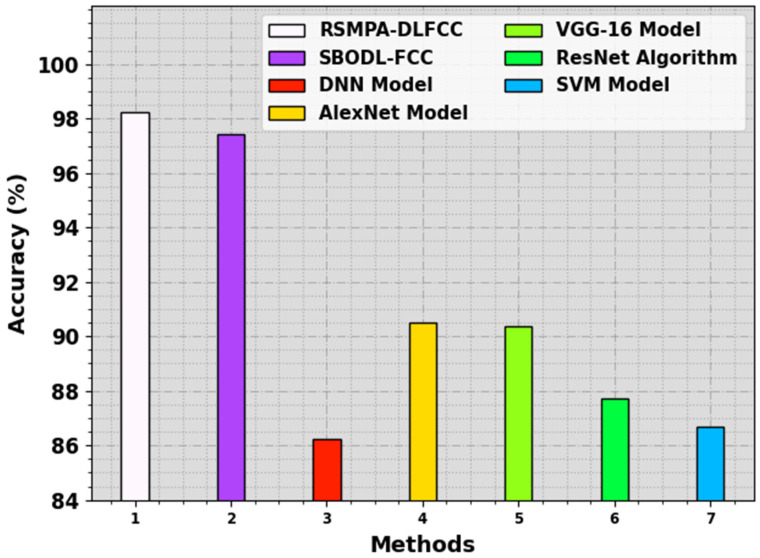
*Accu_y_* Comparative outcome of RSMPA-DLFCC algorithm with other systems.

In Table 3, detailed comparative results of the RSMPA-DLFCC technique are demonstrated with current models [22,23]. Figure 10 investigates a comparative analysis of the RSMPA-DLFCC with recent approaches in terms of $accu_{y}$. The experimental values highlighted that the RSMPA-DLFCC technique reaches an increased $accu_{y}$ of 98.22%, whereas the SBODL-FCC, DNN, AlexNet, VGG-16, ResNet, and SVM models obtain decreased $accu_{y}$ values of 97.43%, 86.23%, 90.49%, 90.35%, 87.70%, and 86.69%, respectively. 

**Figure 11:**
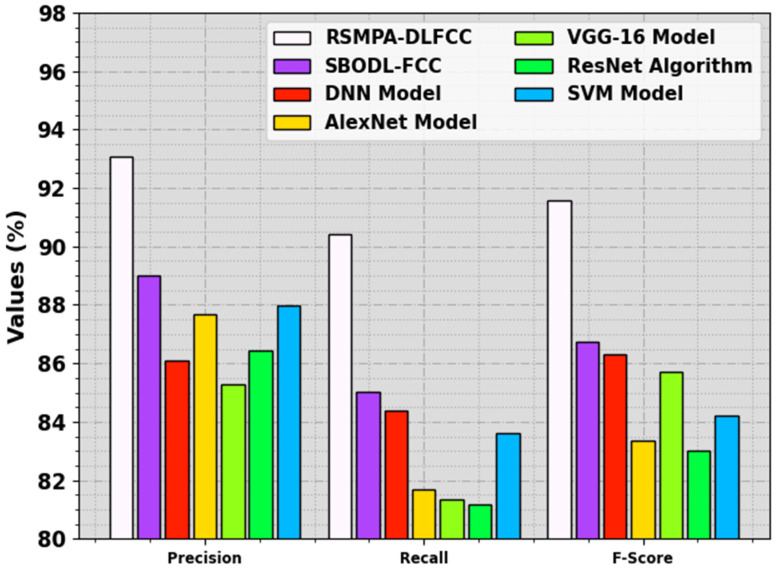
Comparative outcome of RSMPA-DLFCC algorithm with other systems.

Figure 11 investigates a comparative analysis of the RSMPA-DLFCC system with recent techniques, with respect to $prec_{n}$ and $reca_{l}$. The observational data highlighted that the RSMPA-DLFCC system attains a raised $Prec_{n}$ of 93.06%, while the SBODL-FCC, DNN, AlexNet, VGG-16, ResNet, and SVM methods obtain reduced $prec_{n}$ values of 89.02%, 86.11%, 87.68%, 85.28%, 86.42%, and 87.99%, correspondingly. In addition, the RSMPA-DLFCC system attains $reca_{l}$ values of 90.42% whereas SBODL-FCC, DNN, AlexNet, VGG-16, ResNet, and SVM systems get decreased $reca_{l}$ values of 85.03%, 84.39%, 81.7%, 81.35%, 81.18%, and 83.61%, respectively. These experimental data indicated that the RSMPA-DLFCC methodology reaches the maximum food crop classification process.

## 5. Conclusions 

This manuscript offered the development of automated food crop classification using the RSMPA-DLFCC technique. The RSMPA-DLFCC technique mainly investigates the RS data and determines different types of food crops. In the RSMPA-DLFCC technique, the SimAM-EfficientNet model is utilized for the feature extraction process. The MPA is applied for the optimum hyperparameter selection in order to optimize the accuracy of SimAM-EfficientNet architecture. The simulation analysis of the RSMPA-DLFCC method takes place on benchmark UAV image dataset. The widespread result analysis portrayed the higher performance of the RSMPA-DLFCC approach over existing DL models, with a maximum accuracy of 98.22%. In future work, real-time remote sensing data will be a priority, enabling the model to adapt dynamically to changing crop conditions and emerging threats. Moreover, future work can focus on the integration of multi-modal data sources, such as thermal imaging or hyperspectral data, and will broaden the scope of crop classification, providing a more comprehensive understanding of crop health and types. Finally, field tests can be performed to assess the real-world performance and accuracy of the RSMPA-DLFCC technique in diverse agricultural settings and will be essential for its practical deployment and validation.

## Figures and Tables

**Table 1:** Food crop classifier outcome of RSMPA-DLFCC algorithm at 80:20 of TR phase/TS phase.

Class	$Accu_{y}$	$Prec_{n}$	$Reca_{l}$	$F_{score}$	MCC
TR Phase (80%)					
Maize	97.27	94.88	96.57	95.72	93.72
Banana	97.79	95.33	96.31	95.82	94.32
Forest	98.18	94.70	96.02	95.36	94.23
Other	98.31	92.96	92.81	92.89	91.93
Legum	98.90	92.81	87.46	90.05	89.51
Structure	98.28	88.69	75.38	81.50	80.89
Average	98.12	93.23	90.76	91.89	90.77
TS Phase (20%)					
Maize	97.52	95.16	97.74	96.44	94.55
Banana	98.68	96.45	98.03	97.24	96.38
Forest	98.22	95.83	95.47	95.65	94.53
Other	97.98	91.18	89.86	90.51	89.39
Legum	98.60	86.76	86.76	86.76	86.03
Structure	98.29	92.98	74.65	82.81	82.47
Average	98.22	93.06	90.42	91.57	90.56

**Table 2:** Food crop classifier outcome of RSMPA-DLFCC algorithm at 70:30 of TR phase/TS phase.

Class	$Accu_{y}$	$Prec_{n}$	$Reca_{l}$	$F_{score}$	MCC
TR Phase (70%)					
Maize	98.14	96.42	97.87	97.14	95.77
Banana	97.92	94.46	97.57	95.99	94.61
Forest	97.87	94.83	94.40	94.61	93.29
Other	97.70	89.16	91.20	90.17	88.87
Legum	98.16	87.23	79.46	83.16	82.29
Structure	98.07	88.65	71.30	79.04	78.55
Average	97.98	91.79	88.64	90.02	88.90
TS Phase (30%)					
Maize	98.29	97.25	97.41	97.33	96.08
Banana	97.57	93.74	97.24	95.46	93.83
Forest	98.14	95.71	94.69	95.20	94.05
Other	97.62	89.52	90.31	89.91	88.57
Legum	98.50	89.58	81.90	85.57	84.88
Structure	98.29	86.96	79.21	82.90	82.11
Average	98.07	92.13	90.13	91.06	89.92

**Table 3:** Comparative outcome of RSMPA-DLFCC with other systems.

Methods	$Accu_{y}$	$Prec_{n}$	$Reca_{l}$	$F_{score}$
RSMPA-DLFCC	98.22	93.06	90.42	91.57
SBODL-FCC [22]	97.43	89.02	85.03	86.74
DNN l [23]	86.23	86.11	84.39	86.29
AlexNet Model [23]	90.49	87.68	81.7	83.36
VGG-16 Model [23]	90.35	85.28	81.35	85.7
ResNet Algorithm [23]	87.7	86.42	81.18	83.02
SVM Model [23]	86.69	87.99	83.61	84.21

## Data Availability

Data sharing is not applicable to this article as no datasets were generated during the current study.
